# Lower Health-Related Quality of Life in Polytrauma Patients: Long-Term Follow-Up After Over 5 Years

**DOI:** 10.1097/MD.0000000000003515

**Published:** 2016-05-13

**Authors:** Jörn Zwingmann, Paul Hagelschuer, Elia Langenmair, Gerrit Bode, Georg Herget, Norbert P. Südkamp, Thorsten Hammer

**Affiliations:** From the Department of Orthopaedic and Trauma Surgery, University of Freiburg Medical Center, Freiburg, Germany.

## Abstract

Although trauma-associated mortality has fallen in recent decades, and medical care has continued to improve in many fields, the quality of life after experiencing polytrauma has attracted little attention in the literature. This group of patients suffer from persisting physical disabilities. Moreover, they experience long-term social, emotional, and psychological effects that limit/lower considerably their quality of life.

We analyzed retrospective data on 147 polytraumatized patients by administering written questionnaires and conducting face-to-face interviews 6 ± 0.8 years after the trauma in consideration of the following validated scores: Glasgow Outcome Scale, European Quality of Life Score, Short Form-36, Trauma Outcome Profile, and Beck Depressions Inventory II.

Our analysis of these results reveals that polytraumatized patients suffer from persistent pain and functional disabilities after >5 years. We also observed changes in their socioeconomic situation, as well as psychological after-effects.

The rehabilitation of this particular group of patients should not only address their physical disabilities. The psychological after-effects of trauma must be acknowledged and addressed for an even longer period of time.

## INTRODUCTION

The national and international multidimensional concept of health-related quality of life (HRQoL) has 4 components (physical, symptomatic, social, and emotional) and has attracted growing interest in many fields of medicine. HRQoL should be captured and assessed from the patient's perspective.

Beyond the physical wounds and pain associated with surviving a trauma causing multiple injuries, the considerable social and emotional problems these patients often encounter also play a major role. Trauma-associated injuries are the main cause of permanent handicaps especially in young patients, as they compromise their capacity to work and can even lead to complete occupational disability.^1,2^

Although trauma-associated mortality has fallen in recent years and medical care has made continuous progress, HRQoL after surviving polytrauma remains a subject that has attracted little attention in scientific publications. These patients often suffer from persisting limitations in their physical ability to function as well as relevant social and psychological/emotional sequelae.^3–5^ Their lasting physical restrictions also cause serious middle- to long-term socioeconomic damage, especially in patients’ personal and professional lives.^6,7^

Previous examinations of the QoL of patients who have had accidents have yielded disturbing findings. Posttraumatic stress disorder (PTSD) has been diagnosed in 18% to 68% of trauma patients, driving phobias in 38% to 60% of subjects, and anxiety disorders in 8% to 42% and depression in 8% to 45% of trauma patients.^7^ Dittmer (1987)^8^ reported that 48% of the patients in their study cohort had problems at work, that is, 25% financial difficulties and 15% marriage/relationship problems.

In this retrospective investigation by a German university clinic and transregional, certified trauma center, we conducted a long-term examination of the HRQoL and changes in daily life of polytraumatized patients by applying 5 standard and validated scores to be able to demonstrate the tangible, relevant consequences of their accidents.

## PATIENTS AND METHODS

We gathered and assessed retrospective data from a total of 384 polytraumatized patients treated between January 2004 and June 2006 at Freiburg University Clinic. At the follow-up time point, 81 patients (21%) had already died (including 4 suicides). A total of 156 patients could not be included in our examination, and of those, 59% were unreachable because of an address change, 23% never returned the questionnaire despite frequent personal reminders, 12% declined to participate for personal reasons, and 10% stated they were unable to participate for emotional or physical reasons. Thus, we were ultimately able to collect and evaluate the data from 147 patients (49%).

Patient-specific retrospective data of 156 polytraumatized patients were collected from our institution's clinical records, and we conducted oral and/or written questionnaires after 6 ± 0.8 years of the patients validated by the following scores: Glasgow Outcome Scale (GOS),^9^ European Quality of Life (EuroQol) Score,^10,11^ Short Form-36 (SF-36),^12,13^ Trauma Outcome Profile (TOP),^14–17^ and Beck Depression Inventory (BDI) II.^18^

### GOS

The “Glasgow Outcome Score” (GOS) is a scale so that patients with brain injuries, such as cerebral traumas can be divided into groups that allow standardized descriptions of the objective degree of recovery. The first description was in 1975 by Jennett and Bond.

### EuroQoL

EuroQoL (modified Version October 1991: EQ-5D) is a self-rating index instrument expressing health status in a single score. It covers a visual analogue scale and 5 dimensions of health: mobility, self-care, activity, pain/discomfort, and anxiety/depression.^10,11^ EuroQoL is used as a global outcome indicator valuing HRQoL. It determines the presence or absence of quality of life impairing problems without detailed information on the affected domain.

### SF-36

SF-36 is a generic tool for HRQoL measurement containing 36 items grouped in 8 dimensions: physical function, physical role, bodily pain, mental health, emotional role, social functioning, vitality, and general health perceptions.^12,13^ These eight dimensions are summarized into 2 scales: physical and mental health. Norm values based on a representative German sample were adjusted for age and sex adopted according to the underlying distribution in the patient cohort including mainly younger males.^19^ SF-36 Version 2.0 was utilized in POLO-Chart questionnaire.^20^ SF-36 is frequently used to assess quality of life impairments because of different diseases and enables the comparison of quality of life impairments between various entities.

### TOP

TOP is a trauma-specific evaluation instrument(s) for HRQoL assessment. It contains 3 domains: psychosocial domain including 4 scales (depression, anxiousness, PTSD, and social interaction), physical domain including pre- and postinjury pain scales according to 14 different body regions, and functional capacity domain with 3 dimensions: (physical functioning with pre- and postinjury scales according to 14 different body regions [head, neck, shoulder/upper arm, elbow/lower arm, wrist/hand, fingers, thorax, abdomen, spine, pelvis, hip/upper leg, knee/lower leg, food/ ankle, and toes], daily activity status, and mental functioning). The physical functioning scale is a self-reported impairment of function ranging from 0 (no functional deficit) to 10 (no function at all). The pain-reporting scale similarly ranges from 0 (no pain) to 10 (worst pain imaginable). Severe functional deficit and relevant pain was defined as a severity grade ≥5 for at least 1 body region. Additionally, 2 supplements including body image and satisfaction are requested. In total, the TOP consists of 10 different scales ranging from 0 to 100 wherein higher values represent a better quality of life. The TOP has successfully been validated,^21^ and norm values are available from a group with minor injuries. These norm values were used to readjust the 10-scale values in the sense that values of 80 to 100 correspond to normal values (95% of control cases laid in this range).

### BDI

The BDI (BDI, BDI-1A, BDI-II), created by Aaron T. Beck, is a 21-question multiple-choice self-report inventory, one of the most widely used psychometric tests for measuring the severity of depression. Its development marked a shift among mental health professionals, who had until then viewed depression from a psychodynamic perspective, instead of it being rooted in the patient's own thoughts. In its current version, the BDI-II is designed for individuals aged 13 years or older, and is composed of items relating to symptoms of depression such as hopelessness and irritability, cognitions such as guilt or feelings of being punished, as well as physical symptoms such as fatigue, weight loss, and lack of interest in sex. The BDI-II was a 1996 revision of the BDI, developed in response to the American Psychiatric Association's publication of the Diagnostic and Statistical Manual of Mental Disorders, Fourth Edition, which changed many of the diagnostic criteria for Major Depressive Disorder. Items involving changes in body image, hypochondriasis, and difficulty working were replaced. Also, sleep loss and appetite loss items were revised to assess both increases and decreases in sleep and appetite. All but 3 of the items were reworded; only the items dealing with feelings of being punished, thoughts about suicide, and interest in sex remained the same. Finally, participants were asked to rate how they have been feeling for the past 2 weeks, as opposed to the past week as in the original BDI.

Like the BDI, the BDI-II also contains 21 questions, each answer being scored on a scale value of 0 to 3. Higher total scores indicate more severe depressive symptoms. The standardized cutoffs used differ from the original:

1. 0–13: minimal depression
2. 14–19: mild depression
3. 20–28: moderate depression
4. 29–63: severe depression.

A statement of approval by an Ethical Committee was not necessary. The patients’ various epidemiological and injury-specific data (severity and location) were tested via IBM SPSS Statistics 22 (2013) and Microsoft Excel 2010 for significance in individual scores and subjected to univariate analysis of variance and the Mann-Whitney *U* test.

## RESULTS

### Lethality

At follow-up, 81 patients (21%) were deceased. Cause of death in 49 patients was the accident itself or its sequelae. We were unable to obtain valid cause-of-death data on 17 patients. Six patients died of an accident-unrelated cause (heart attack, murder, etc), and 5 died in a nursing home in which we could not determine the extent to which the death was causally related to the original accident. Four patients had committed suicide before follow-up.

We collected data from 147 patients (49%) aged an average 40 ± 19 years; 75% of the patents were male and 25% female. They underwent a follow-up after an average of 6 ± 0.8 years. The average Injury Severity Score (ISS) was 28 ± 11—quite high, and reflecting our cohort of polytraumatized patients and the severity of their injuries treated at a maximum-care German trauma center such as ours.^22,23^ Length of hospital stay was on average 22 ± 14 days; average stay on the intensive care unit (ICU) was 7.8 ± 7.5 days. The cause of trauma was in 68.8% a road accident, whereby 29.3% were passengers in a car, 25.2% of the injured were motorcyclists, 8.2% cyclists, and 6.1% pedestrians. In 23.1%, the injury had been caused by a fall from a height of >3m, and 8.2% suffered traumas from causes other than those listed above (Table 1).

**TABLE 1:**
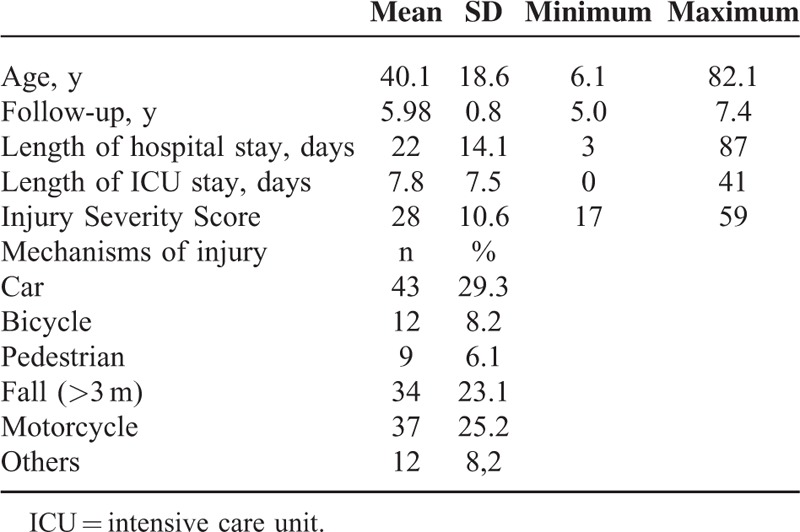
Epidemiological Data of 147 Analyzed Patients (75.5% Male/24.5% Female)

### GOS

**FIGURE 1:**
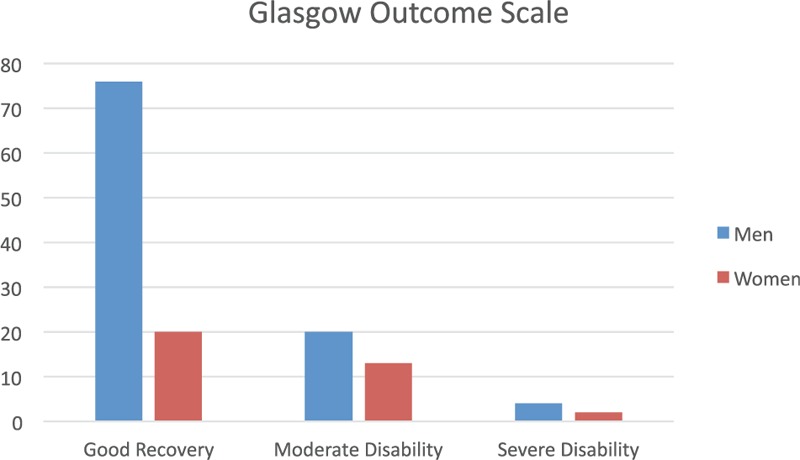
Illustrates the Glasgow Outcome Scale of the men and women by percentage in our study cohort.

The GOS revealed in 71.1% of patients a value of 5 and thus no or minimal neurological or psychological deficit. The GOS in 22.9% of patients was 4, reflecting as long-term sequelae a moderate handicap not requiring everyday assistance. However, 5.6% of our patients had GOS score of 3, revealing severe damage and a permanent need for assistance in daily life. We identified no patient in a persistent vegetative state during our retrospective investigation (one can speculate as to whether there were some among those we could not contact). Figure 1 illustrates the GOS of the men and women by percentage in our study cohort.

### EuroQol Score

**FIGURE 2:**
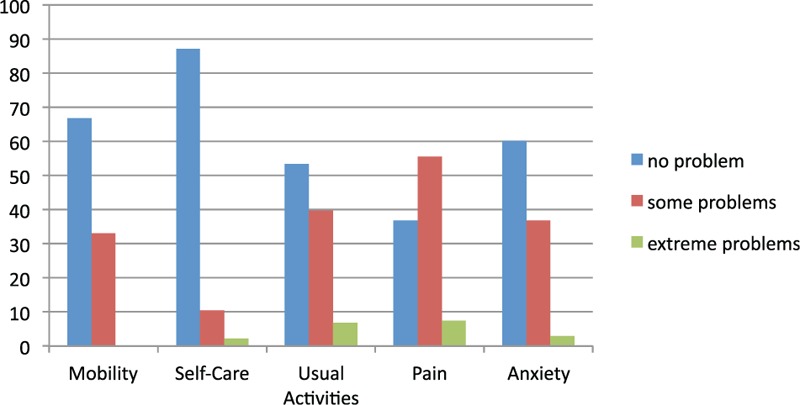
Health-related quality of life according to the EuroQol Score.

HRQoL according to the EuroQol Score is shown in Figure 2. In short, 6.8% of the patients have difficulty or pain in daily activities, and for 7.5%, the difficulties are extreme. In the categories mobility, general activities, pain, and anxiety, over one-third to half of all patients (33.1%–55.6%) have problems. The vast majority of patients have no problems with personal hygiene (87.2%): 51.5% of patients had an index of ≤0.8, and 18.7% even had a value ≤0.5.

Our statistic assessments revealed no association between the severity of the injury and its location (AIS lower and upper extremity, severity of craniocerebral injury). Age, however, did demonstrate a significant effect on the EuroQol score (*P* < 0.001).

### SF-36

We compared the analyzed data from our SF-36 QoL questionnaire results with those age- and sex-adjusted values from a representative-norm German population.^19^ This (physical [score 46.2] and mental [score 46.1] health) reveals mildly reduced values in comparison to a representative German sample, which were adjusted for age and sex adopted according to the underlying distribution in the patient cohort including mainly younger males (score 50). The mental health revealed significant correlations between the severity of a craniocerebral injury as measured via the Abbreviated Injury Scale (AIS) Head (*P* *=* 0.032), as well as with BDI II values (*P* *<* 0.001) and the loss of (or change in) job (*P* *=* 0.014). Regarding the physical health, there was a significant correlation with age (*P* *<* 0.001) and patients with and without injuries to the lower extremities (*P* *=* 0.015).

**FIGURE 3:**
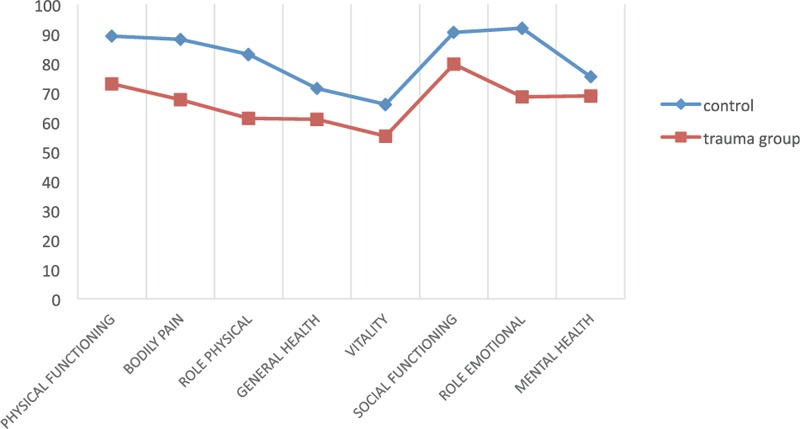
Our study patients’ average values in the Short Form-36 questionnaire's 8 domains in direct comparison with the norm population.

Figure 3 shows our study patients’ average values in the SF-36 questionnaire's 8 domains in direct comparison with the norm population: The greatest discrepancy was found in the physical and emotional role function.

The domain physical pain revealed a significant correlation in patients with severe injuries to the lower extremities with an AIS ≥3 (*P* = 0.031). Age appeared as a significant factor in physical (*P* *<* 0.001) and emotional (*P* *=* 0.043) role functions. A total of 67.4% of our patients claim to suffer injury-related pain on an everyday basis.

### TOP

**FIGURE 4:**
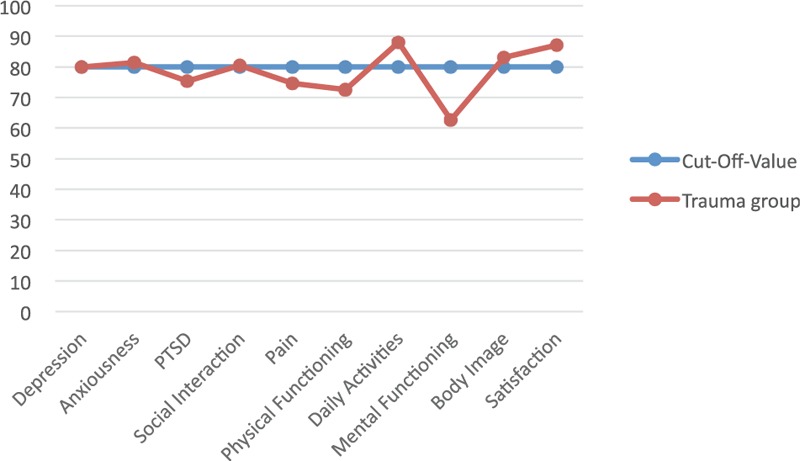
The threshold value of trauma-specific quality of life as reflected in the Trauma Outcome Profile scores of healthy individuals is defined as ≥80 for each domain.

The threshold value of trauma-specific QoL as reflected in the TOP scores of healthy individuals is defined as ≥80 for each domain and is illustrated in Figure 4. We noted impairments in the domains PTSD, pain, and physical and mental function. In the mental function domain, we observed a significant correlation with age (*P* *=* 0.016) and the severity of craniocerebral injury, evident in the AIS Head (*P* *=* 0.02). Moreover, age was a significant parameter in the pain domain (*P* *=* 0.017). PTSD was diagnosed in 42.2% of our study patients.

### BDI II

**FIGURE 5:**
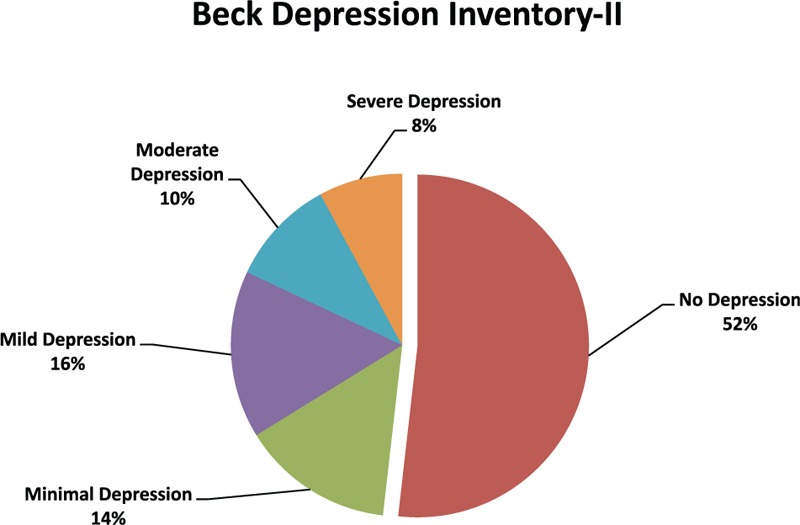
Illustrates the Beck Depressions Inventory II-measured severity of depressive symptoms.

Figure 5 illustrates the BDI II-measured severity of depressive symptoms, wherein 48% of our cohort displayed peculiarities and signs of depressions. The AIS revealed the craniocerebral injury's severity to be a significant parameter (*P* *=* 0.034).

### Job Changes, Perceived Financial Loss, and Marriage/Partnership Status

Seventy-six percent of our patients stated there had been no change in their job situation after their injury. At follow-up, 16% had become unemployed, 3% had changed jobs, and 2% had undergone training for a new job. We had no access to job data from 3% of the study patients. Comparing the jobless with those with no change in job situation revealed much lower values in the former in all SF-36 and TOP domains.

According to their self-reported feedback at follow-up, 65% of our patients had suffered no financial losses from their injuries or the consequences thereof, 29% had suffered financial losses in their opinion, and 6% the patients did not want to answer this question. The comparison between those patients who suffered financial losses with those who had not revealed much lower values in the former group in all the SF-36 domains and except for daily activities in all the domains in the TOP.

At follow-up, 76% of our patients had experienced no change in their marital/partnership status, 16% were living alone after the accident and in a relationship, and 8% said they had been in a relationship before the accident but were living alone and single without a steady partner.

## DISCUSSION

The aim of this long-term follow-up investigation was to describe and analyze HRQoL in all its components in patients who had been severely injured. QoL was defined as a new endpoint in post-trauma patients in contrast to the standard morbidity and mortality 10 years ago.^24^

The personal perspectives of patients play a particularly important role in the context of injuries experienced during an accident, as they are often associated with substantial impairments in patients’ physical and emotional well-being. It is only through the affected patient's perspective that we can capture precisely enough and adequately assess persisting negative effects such as consistent physical pain, functional impairments, physical handicaps or disfigurations, and emotional problems or shortcomings in one's social life or ability to participate in activities. The fact that, thanks to the enormous progress recently made in the therapy of severe injuries and the rise in the numbers of survivors of the most horrible accidents, the need to capture parameters measuring self-reported health is now higher rather than lower.^25^

When examining typical polytraumatized patients in a maximum-care clinic in Germany, one is dealing with mostly young, predominantly male patients. As this retrospective is monocentric, it has several limitations. In particular, there is an indisputable bias because some patients could no longer be reached, or could (or would) not participate in the follow-up. The enrollment level we achieved of 49% of patients in our long-term follow-up who could be examined resembles the enrollment levels cited in similar studies with even briefer follow-up periods.^26–28^ Compared with other investigations, our patients’ scores are similar to or even better than those in other patient cohorts.^16,28^ Moreover, potential preexisting psychiatric history has not been collected, which could be a bias.

Our patients’ SF-36 values reveal an impaired QoL in patients who have suffered multiple injuries. Although this questionnaire is a recognized measuring instrument nationally and internationally, we draw attention to the fact that it provides information on HRQoL. It does not address the specific problems of any one patient group. We therefore employed additionally the TOP score to address the specific problems of severely injured patients. Together, and as they address different domains, the SF-36 and TOP scores provide detailed information on the kind of impairment in everyday life. All the domains in our patients’ SF-36 were lower than those in an age- and sex-adjusted norm population. The greatest discrepancy in physical ability to function and physical and emotional role function is also reflected in their TOP scores, wherein the PTSD domain was much lower.

Our cohort's TOP score revealed no deviation from the norm in the depression and anxiety domains, although we did note peculiarities in the PTSD category, even after >5 years after their injury. The incidence of PTSD in such patients cited in the literature is 18% to 42%.^29^

A serious problem, especially for polytraumatized patients, is the potential loss of or forced changes in their job and the socioeconomic ramifications thereof. Of our patients, 29% reported that they had suffered a financial loss after the accident. This percentage is very high, although still higher ones (up to nearly 50%) are reported in the literature. Seventy-six percent of patients of our cohort were able to continue working at their original job, others had to change jobs or train for a new one, and 16% were unemployed. Some working groups have cited rates as high as 33% of severely injured patients who have lost their jobs.^6,30,31^

HRQoL has become a key criterion when assessing results in many fields of medicine. It supplements the traditional clinical and social-medical criteria by providing the patient's perspective, thereby enabling a more rounded, thorough, and practical evaluation of the benefit of medical interventions. Such an expanded assessment is particularly appropriate for traumatology, as accident injuries can be associated with substantial psychosocial drawbacks for the affected patients.

## CONCLUSION

Our findings reveal that even after >5years, polytraumatized patients still suffer from persisting pain and functional impairments. We also demonstrate that negative socioeconomic effects were associated with emotional repercussions. When rehabilitating these patients, it is not enough to just treat the injuries’ physical symptoms. Their emotional consequences should also be identified, as they are apt to benefit from long-term therapy.
